# Bringing Order to Chaos in High-Entropy Electrocatalysts

**DOI:** 10.1021/acsenergylett.6c00141

**Published:** 2026-02-16

**Authors:** Jing Yu, Ren He, Neus G. Bastús, Jordi Arbiol, Andreu Cabot

**Affiliations:** † 235241 Catalonia Institute for Energy Research (IREC), Sant Adrià de Besòs, 08930, Catalonia, Spain; ‡ 231882 Catalan Institute of Nanoscience and Nanotechnology (ICN2), CSIC and BIST, 08193, Barcelona, Catalonia, Spain; § ICREA, Pg. Lluis Companys, 08010 Barcelona, Catalonia, Spain

## Abstract

High-entropy materials (HEMs) have emerged as a transformative platform for electrocatalysis. Their appeal lies in the vast compositional versatility enabled by the combination of five or more elements, which generates a rich diversity of atomic configurations and surface sites ideally suited for complex multistep reactions. Recent years have witnessed explosive growth in the development of HEMs across diverse material classes and their application to a wide range of electrochemical reactions. Yet significant challenges remain to fully harness their capabilities while managing their intrinsic structural and chemical complexity. Advancing the field requires exploring compositional space, pinpointing reaction sites, and achieving atomic-level control of surface composition and organization. Much remains to be done, calling for breakthroughs in materials design, characterization, and synthesis strategies and technologies. Ultimately, as highlighted here, beyond electrocatalytic applications, HEMs embody a new paradigm in materials discovery, linking precise engineering, correlative multimodal characterization, and high-throughput experimentation and computation.

## Graphical abstract

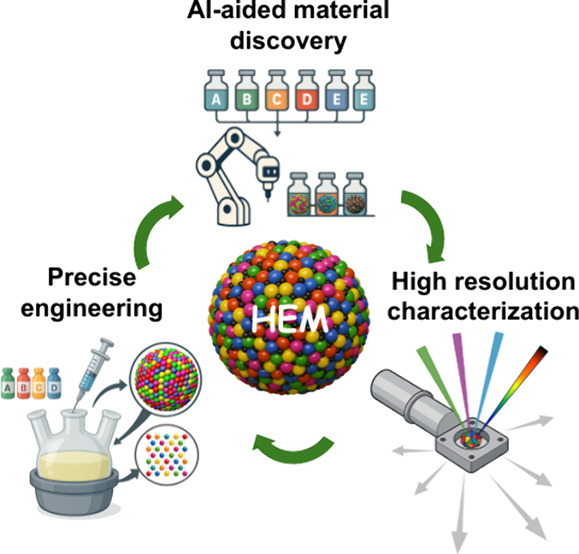

**H**igh entropy materials (HEMs) are generally defined as single-phase compounds containing five or more principal elements. The term and definition trace to Yeh et al.’s 2004 seminal paper, which framed the configurational entropy (ΔS_conf_) contribution in the Gibbs free energy (*ΔG* = *ΔH* – *TΔS*) as a potential stabilizer of multielement solid solutions. Specifically, they argued that in equiatomic mixtures of five principal elements, the configurational entropy could be large enough at synthesis temperatures for the *–TΔS*_conf_ contribution to offset the formation enthalpies of competing intermetallic phases, thereby favoring a single-phase solid solution.

While subsequent work indicates that entropy is rarely the sole or even dominant driver of formation, stabilization, or properties, the “high-entropy” label has taken root and broadened well beyond metallic alloys to encompass oxides,^1,2^ chalcogenides,^3^ nitrides,^4,5^ phosphides/phosphates,^6,7^ carbides, borides,^8^ halides,^9^ MXenes,^10^ metal–organic frameworks,^11^ polymers, and even single-atom-decorated systems.^12,13^

Originally explored for mechanical performance, HEMs have since shown promise across numerous energy conversion and storage fields, ranging from nuclear reactor walls to thermoelectricity, batteries, and electrocatalysis.^14–19^ Whether entropy itself governs performance remains debated, but combining ≥ 5 elements undeniably opens a vast compositional and structural design space that can be exploited to meet application-specific targets using abundant, low-toxicity constituents. For perspective, selecting 5 elements from a pool of 40 usable elements of the periodic table already yields 658,008 unique quinary combinations, and summing all choices from 5 to 10 elements exceeds 1.2 billion possibilities. The total number of combinations using 5 to 40 useful elements is about 1.1 trillion (10^12^), while allowing variable stoichiometries renders the space effectively unbounded, as Cantor noted back in 2014 (Figure 1).^20^

**Figure 1:**
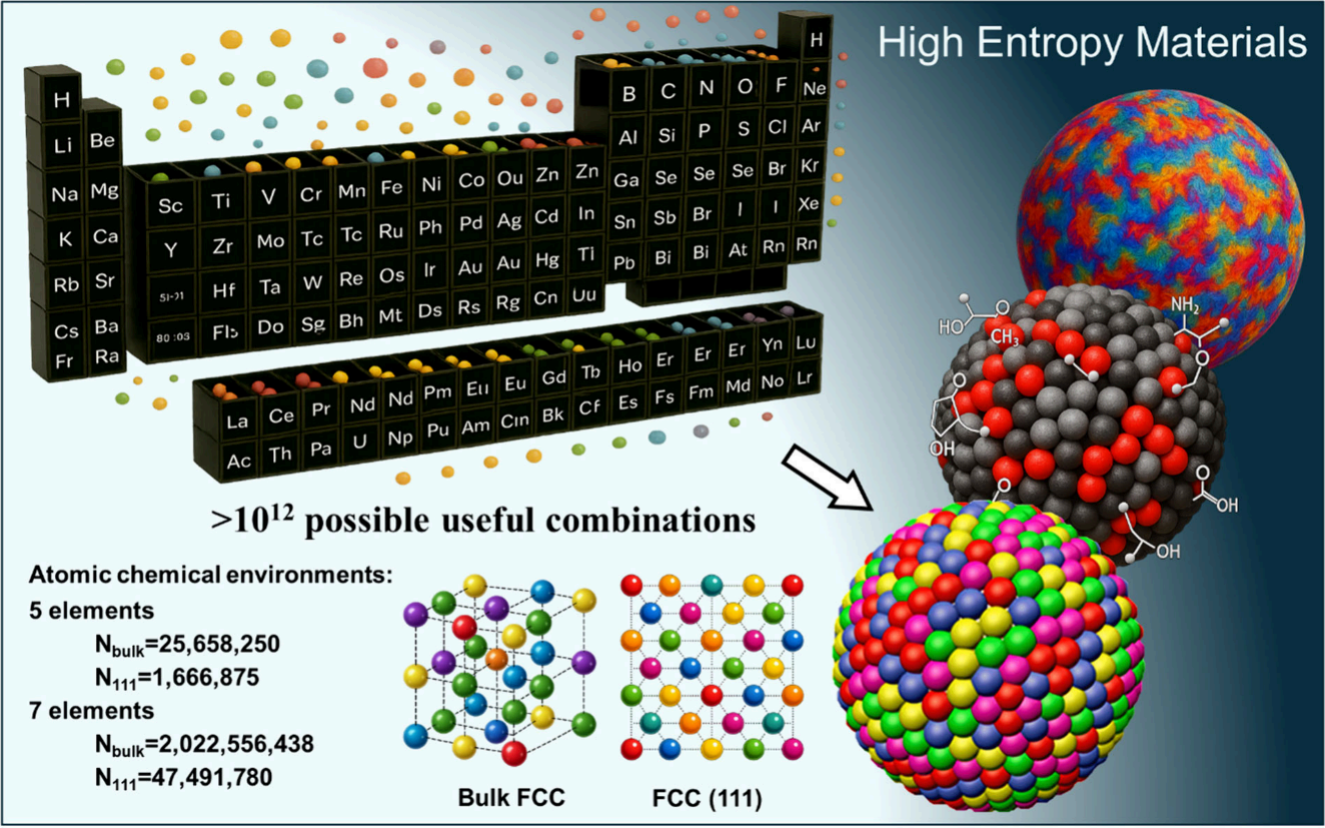
HEM immense composition space and even vaster diversity of atomic chemical configurations. Schematic illustration of how mixing multiple principal elements generates a huge range of potential combinations (10^12^, considering 40 useful elements), each of them offering a large number of distinct bulk (*N*_bulk_) and surface (*N*_111_) atomic environments as calculated considering an FCC crystal structure, taking into account symmetries and considering only nearest neighbors.

Whether entropy itself governs performance remains debated, but combining ≥5 elements undeniably opens a vast compositional and structural design space that can be exploited to meet application-specific targets using abundant, low-toxicity constituents.

Beyond the huge number of possible element combinations, even a single HEM composition yields an enormous set of surface site configurations. In a face-centered cubic (FCC) high-entropy alloy (HEA) with five elements and random occupancy, considering only the first coordination shell (12 nearest neighbors), and accounting for lattice symmetries, the number of different local chemical environments for a given central atom exceeds five million. Allowing the central atom to vary among the five elements raises this to >25 million distinct nearest-neighbor configurations, with a seven-element HEA exceeding ∼2 billion. On a (111) surface, where coordination is reduced, there remain >300,000 distinct surface configurations per element, totaling above 1.5 million for a quinary system and nearly 50 million for a septenary (Figure 1).

This abundance of distinct local chemical environments creates an exceptionally broad spectrum of surface sites, yielding tunable quasi-continuous distributions of adsorption energies. Such distributions can lower reaction overpotentials and are particularly well suited to multistep reactions that require balanced binding across several intermediates. Conversely, this diversity may compromise selectivity, though notable reports do also claim improved selectivity with carefully tailored HEM catalysts.

Beyond compositional and surface-site diversity, multiple disordered elements can reshape the electronic structure, with concomitant changes in electron scattering, band broadening, and spectral-weight redistribution around the Fermi level (*E*_F_). Because the atomic potential is intrinsically nonperiodic in random multielement solids, electronic states exhibit finite lifetimes and momentum/energy broadening, yielding smeared band features and a broadened density of states (DOS) near *E*_F_ compared with ordered counterparts.^21^ Consequently, HEM surfaces are better described by distributions of local electronic descriptors (e.g., d-band center, bandwidth, adsorbate–metal hybridization), which translate into quasi-continuous spreads of adsorption strengths and activation barriers that tune not only chemisorption energies, but also kinetics, and can, in multistep transformations, shift rate-determining steps and potentially pathway preference. This electronic heterogeneity can couple to local strain/stress, so atomic-level compressive/tensile environments reflect not only size mismatch but also environment-dependent charge redistribution, linking ensemble chemistry to site-to-site variations in adsorption and reconstruction.^22^

Configurational disorder also generates strong local lattice distortions and a heterogeneous energy landscape in which defect formation and migration barriers become distributions rather than single values. Such rough landscapes can suppress defect mobility through correlated/backward motion, while emerging short-range order can introduce local trapping/pinning, thereby influencing reconstruction, segregation, coarsening, self-healing, partial reversibility of defect/segregation states under cycling, and the stabilization of metastable states.^23,24^

Beyond incremental tuning within a fixed state manifold, multimetal and particularly HEM compositions can expand the accessible manifold of active states. By altering local ligand fields, covalency, and redox energetics, neighboring elements can stabilize oxidation states, coordination motifs, or adsorbate configurations of a primary active element that are not reachable (or not persistent) on the single-metal analogue under the same conditions, opening alternative, lower-barrier pathways. This is particularly transparent for OER, where increased metal–oxygen covalency and redox reach can promote oxygen participation and facilitate a shift from an adsorbate evolution mechanism toward a lattice oxygen mechanism. More broadly, the same principle applies whenever local ensembles stabilize otherwise inaccessible reactive intermediates or charge states.^25^

These electronic-structure effects are coupled to transport. The random combination of several elements can modify electronic conductivity through multiple, sometimes competing, mechanisms: (i) changes in band filling and hybridization that shift carrier density and effective masses; (ii) modified defect chemistry, e.g., vacancies, mixed valence, or dopant-like states, that alters carrier concentration; (iii) microstructural stabilization that can either promote or hinder macroscopic conduction pathways; and (iv) enhanced disorder-driven scattering that increases resistivity and can reduce mobility, particularly in metallic or semimetallic systems. Thus, HEMs span regimes from metallic to insulating.

In metallic HEMs, severe chemical disorder can push transport toward the Mott–Ioffe–Regel limit, where mean free paths become extremely short and near-*E*_F_ electronic features are strongly smeared.^26,27^ In this highly disordered, diffusive regime, disorder can also amplify electron–electron interaction effects, often discussed in the appropriate, frequently low-temperature regime, yielding a partial suppression of the DOS at E_F_ and non-Drude conductivity corrections. Together, these effects imply that some nominally metallic HEMs may exhibit transport-limited behavior with reduced effective low-energy electronic states, so apparent electrocatalytic kinetics can become constrained by charge delivery in realistic catalyst layers.^28,29^

In contrast, many HEMs are insulating or hopping-dominated because disorder promotes carrier localization, and nominal aliovalent substitution can be compensated by redox/defect chemistry rather than generating free carriers.^30^ In such semiconducting/localized HEMs, interaction effects can manifest as a soft Coulomb gap with Efros–Shklovskii-type hopping transport in the appropriate regime, while the same heterogeneous energy landscape and emerging short-range order can further modulate defect kinetics, diffusion, and reconstruction pathways.^31,32^ Consequently, transport in HEMs is not captured by a single universal descriptor and may improve or deteriorate depending on composition, phase, defect populations, and microstructure.

Transport can strongly shape apparent electrocatalytic performance, reaction pathways, and stability. Poor electronic conductivity or charge percolation increases ohmic losses within catalyst layers, raises the apparent reaction overpotential, and can shift measured behavior from intrinsic charge-transfer control toward transport-limited regimes, especially in thick electrodes, at high current densities, and for semiconducting HEM classes. These transport characteristics, together with ionic conductivity and double-layer capacitance, govern local overpotentials, current distribution, and the ability to sustain high reaction rates without severe concentration or ohmic losses, particularly in thick films or porous architectures. Spatially nonuniform charge transport can also generate local potential gradients that concentrate reaction inhomogeneously, bias pathways, and accelerate degradation via hot spots, extreme local pH, or nonuniform surface reconstruction. The coupling between electronic structure and transport can also influence stability: localized states or weakly bonded constituents may be preferentially oxidized or leached under bias, dynamically reshaping the active surface and its catalytic response. Conversely, improved transport and stable percolation networks enhance active-site utilization, homogenize current distribution, and mitigate conditions that drive dissolution, passivation, and mechanical failure. Therefore, mechanistic interpretation should explicitly separate, and where possible quantify, intrinsic surface reactivity from bulk/mesoscale transport contributions.

Accordingly, apparent catalytic trends can reflect intrinsic kinetics intertwined with transport limitations and defect-driven evolution (reconstruction, segregation, coarsening, self-healing, and/or partial reversibility under cycling) that depend on microstructure, phase stability, percolation, and operating history. This requires pairing catalytic descriptors with transport-aware testing. Conductivity measurements and electrochemical impedance spectroscopy can distinguish whether performance changes originate from charge transfer, mass transport, or electronic percolation, while benchmarking under conditions that suppress ohmic artifacts (thin films, low loadings, controlled electrode architectures) helps isolate intrinsic kinetics. Ultimately, linking disorder-induced changes in DOS/transport to kinetic metrics and degradation signatures is essential to move from descriptive correlations to predictive, mechanism-based control of activity and durability.

The colossal search space also renders purely sequential, trial-and-error optimization impractical; synthesizing one composition at a time with a single optimized protocol cannot map the HEM landscape toward any specific performance target. Yet several HEM electrocatalysts have been reported to promote a wide range of electrocatalytic reactions,^17^ including CO_2_,^33–35^ oxygen,^36^ nitrogen and nitrate reduction;^37,38^ oxygen and hydrogen evolution;^1,3–5,8,11^ and various oxidation reactions,^39,40^ as well as battery-relevant processes such as lithium polysulfide conversion in Li–S batteries and oxygen reactions in metal–air/oxygen batteries,^2,7,41–45^ often without systematic exploration of adjacent compositions.

Beyond any specific material class or reaction, unlocking the huge potential of HEM electrocatalysts requires further development of three interconnected capabilities. First, atomic-level synthetic control that enables deliberate, reproducible, and scalable tuning of surface and near-surface composition and organization. Second, correlative multimodal characterization capable of (i) resolving atomic distributions ex situ, and (ii) capturing surface reconstruction dynamics and active sites in situ/operando, linking real working surfaces to performance. Third, high-throughput experimental and computational exploration to screen compositions and process variables, identify stable configurations under operating conditions, and derive descriptors that link performance with material and processing parameters. Together, these capabilities should enable mechanism-aware design rules and accelerate the emergence of optimized, application-ready electrocatalysts among the immense range of HEMs.

Effective optimization of HEMs, including metal alloys, oxides, chalcogenides, phosphides, etc., for specific reactions first requires the ability to synthesize single-phase compositions containing all target elements. In this direction, it is often assumed that entropy plays a central role in stabilizing the multicomponent phase. From the Gibbs free-energy relation, *ΔG = ΔH* – *TΔS*, it is then frequently inferred that high temperatures are required to form HEMs. This reasoning has driven the development and widespread use of rapid thermal processes, such as carbothermal shock, and high-energy methods like laser ablation to produce HEM nanoparticles or films for electrocatalytic applications.

Rapid thermal processing offers two advantages: fast quenching to lock in the high-temperature solid solution and ultrashort heat exposure to suppress particle growth, thereby maximizing surface area and interaction with electrolyte. However, it affords little control over HEM particle size, facet exposure, and atomic organization, constraining surface engineering. Consequently, alternative low-temperature routes are needed to stabilize phases under milder conditions with improved morphological and compositional control.

Beyond entropy-dominated high-temperature routes, kinetics can also be used to produce HEMs at near-ambient temperature, enabling finer control over HEM parameters. Kinetics-driven syntheses exploit the slow diffusion and limited long-range atomic mobility, preventing long-range atomic redistribution and phase separation. With monomer-level homogeneous mixing, e.g., within a liquid or gas phase or via concurrent mechanochemical processing or solids, and with mass transport limited to short length scales, the system can be trapped in a metastable, yet durable, multielement solid solution.^46^

Besides entropy and kinetics, we recently argued that enthalpy contributions, particularly surface and interface energies, can also drive HEM stabilization at the nanoscale.^46^ HEM disaggregation into multiple phases generates additional surfaces/interfaces with high energy cost. As particles shrink, this penalty dominates, defining a critical radius below which a single-phase HEM is thermodynamically favored over multiphase configurations. Figure 2a plots the Gibbs free-energy change for a single-phase nanoparticle segregating into either two wetting or two nonwetting phases as a function of particle size. A critical size appears below which the single phase remains stable, determined by the trade-off between per-atom HEM cohesive energy and the surface/interfacial penalties incurred upon segregation, which generally depend on relative atomic size, electronegativity, preferential valence, and crystal structure.

**Figure 2:**
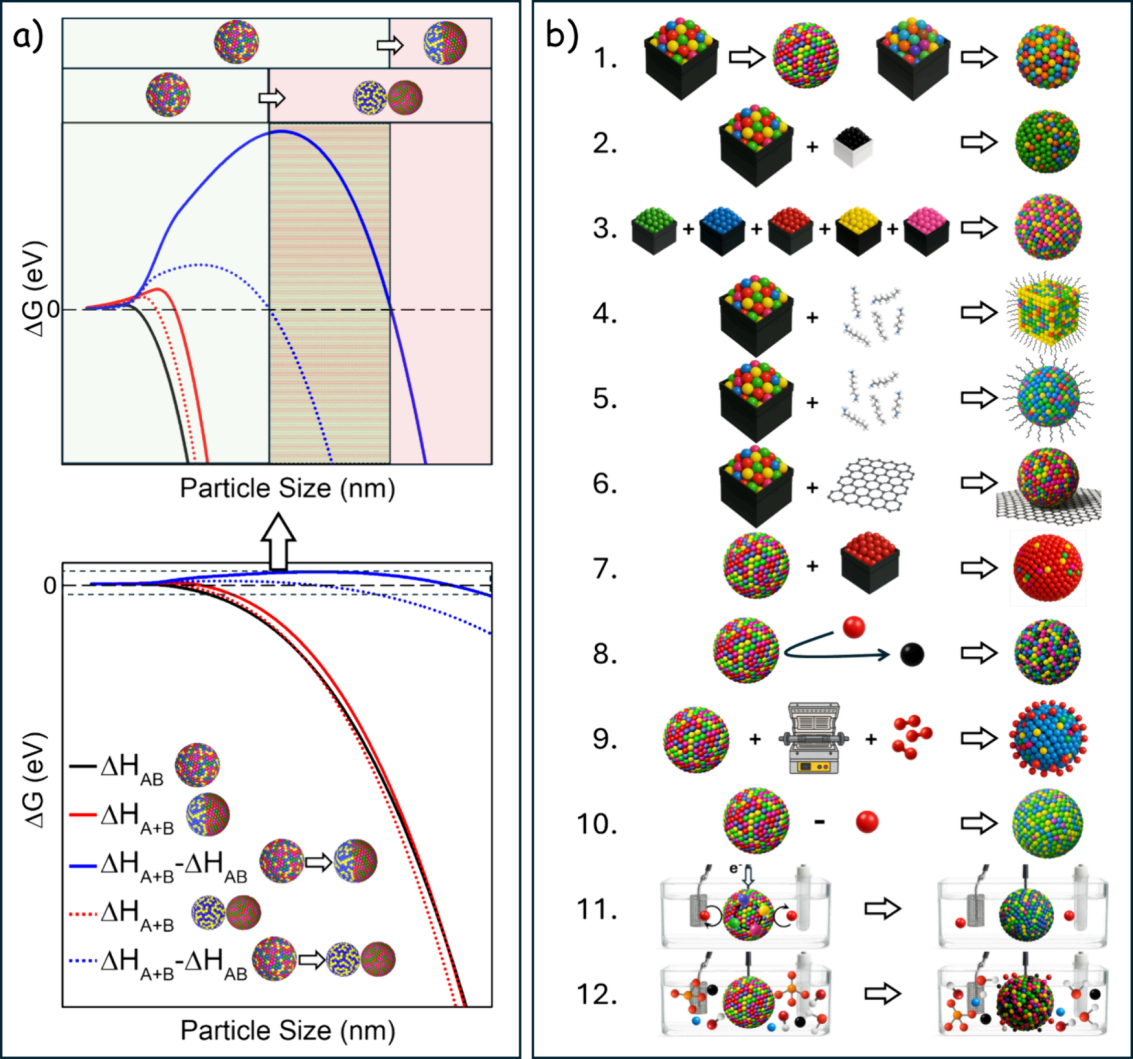
Pathways to single-phase HEM nanoparticles with controlled surface composition. a) Gibbs free energy change as a function of particle radius for the phase segregation of a spherical HEM nanoparticle into two hemispherical (wetting) and two spherical (nonwetting) domains. Only the enthalpy of each phase is considered. We assume the enthalpy per atom in the segregated phases is 15% lower than in the HEM and that the interface and surface energies are 0.4 eV. With these values, the critical particle sizes are 5.5 nm (wetting) and 3.5 nm (nonwetting). b) Scheme of different strategies to adjust HEM surface composition and atomic organization during the synthesis step (1–6), a posteriori (7–10) and in operation conditions (11–12): 1) Selection of element type and concentration; 2) Addition of dopants; 3) Sequential addition/reaction of precursors; 4) Adjusting particle facets, e.g. by means of surface ligands; 5) Leveraging element-selective binding of surface ligands; 6) Selection of the support material; 7) Postsynthesis coating; 8) Atomic replacement; 9) Thermal or plasma treatments in controlled atmosphere; 10) Selective leaching/etching; 11) Electrochemical activation under controlled current/voltage; and 12) Adjusting electrolyte composition.

Since catalysis depends on the outermost layers, stabilizing a single-phase HEM is not enough; fine control of surface composition and atomic order is crucial for optimizing electrocatalysis. While HEMs are often modeled as random solid solutions, preferential short-range order likely develops from element–element affinities and environmental interactions.^47,48^ Because the surface of HEMs, including metal alloys, oxides, chalcogenides, phosphides, etc., experiences a chemical environment distinct from the bulk, market deviations in composition and atomic organization arise and can evolve under processing, postprocessing, and operating conditions. For example, in an HEA exposed to oxygen, oxophilic elements segregate to the surface. In fact, the vast configurational manifold and insignificant energy differences between configurations render HEMs not static solids but highly mobile, adaptive catalytic systems whose surface and near-surface composition, atomic arrangement, and reactivity continuously evolve with temperature, atmosphere, electrochemical potential, and adsorbates, factors that can reorder which elements occupy the top layer and how they are arranged.

The vast configurational manifold and insignificant energy differences between configurations render HEMs not static solids but highly mobile, adaptive catalytic systems whose surface and near-surface composition, atomic arrangement, and reactivity continuously evolve with temperature, atmosphere, electrochemical potential, and adsorbates, factors that can reorder which elements occupy the top layer and how they are arranged.

These sensitivities can be exploited to deliberately direct surface composition and atomic organization, thereby tuning performance. In practice, the real operating configuration reflects a balance between thermodynamic and kinetic drivers across synthesis, post-treatments, and operation. This balance can be adjusted through several specific levers (Figure 2b).

At the synthesis step (panels 1–6 in Figure 2b), the selected elements and their relative elemental ratios set the baseline ensemble of possible atomic configurations, while elemental affinities dictate preferred nearest neighbors, relations that can be probed with *ab initio* calculations. Within a given HEM formulation, trace amounts of additional elements, i.e., dopants, can be used to stabilize specific atomic organizations. Beyond the preselected composition, the synthetic pathway strongly influences outcomes: the introduction sequence and the relative reactivity and coordination chemistry of precursors, which can be adjusted via redox additives and coordinating agents, govern growth kinetics and surface stoichiometry. Particle morphology provides another design lever, as different facets likely stabilize distinct atomic configurations. Surface ligands that bind selectively can both direct faceting, indirectly tuning surface composition, and directly adjust surface stoichiometry and ordering through element-specific binding affinities. Careful ligand selection is thus crucial to balance structure-directing benefits against potential catalyst poisoning by strongly bound species. In supported catalysts, strong metal–support interactions, lattice mismatch, and induced strain can also redistribute which elements remain surface-exposed versus buried in the bulk or at interfaces.

Postsynthesis (panels 7–10 in Figure 2b), surface chemistry can be adjusted with submonolayer precision using chemical, electrochemical, or physical coating methods. Coupled with brief anneals, these compositional modifications can extend several nanometers below the surface, creating graded profiles. Ion-exchange and galvanic replacement can selectively substitute one or more specific elements in the surface and subsurface. Thermal and plasma treatments can further refine the surface structure and composition: e.g., vacuum or inert anneals promote segregation of low-surface-energy elements, whereas reactive anneals in O_2_, H_2_, S/Se, P, or halogen atmospheres drive selective enrichment and ultrathin compound shells with thickness and stoichiometry tunable via temperature and partial pressure. Even under ambient conditions, adsorbates such as CO, H_2_, O_2_, halides, or chalcogen species can drive selective surface enrichment by attracting specific elements to the top layer. Additionally, selective leaching/etching can partially or completely remove target elements, potentially exposing a more active configuration by enriching the surface in others.

Finally, beyond synthesis and post-treatment, the working environment itself actively participates in shaping the catalyst surface (panels 11 and 12 in Figure 2b). Applied voltages, redox currents, and the electrolyte composition, encompassing pH, additives, reactants, intermediates, and products, continuously drive surface reorganization. This can be steered by activating the HEM catalyst under controlled electrochemical conditions and/or introducing targeted additives to stabilize desired configurations in operation. Thus, genuine control of HEM functionality requires not only mastery of synthesis and post-treatment, but also a deep understanding of the dynamic feedback between surface evolution and reaction environment.

Genuine control of HEM functionality requires not only mastery of synthesis and post-treatment, but also a deep understanding of the dynamic feedback between surface evolution and reaction environment.

Ultimately, the most effective strategy for tailoring surface composition is system-specific and must be established case by case. Equally critical is assessing scalability, throughput, batch-to-batch reproducibility, environmental footprint, safety, and cost, so that atomic-level control achieved in the lab translates to production-relevant contexts. Striking the right balance between precision and practicality will determine how rapidly tailored HEM catalysts move from fundamental studies to real-world electrochemical devices.

In addition to highly precise chemical engineering, achieving this level of compositional control in HEMs, including metal alloys, oxides, chalcogenides, phosphides, etc., requires feedback from atomic-resolution and surface-specific characterization tools (Figure 3). Atomic-scale real-space mapping by aberration-corrected scanning transmission electron microscopy (STEM) coupled with energy-dispersive X-ray spectroscopy (EDS) or electron energy-loss spectroscopy (EELS), including energy-loss near-edge structure (ELNES), is typically used to demonstrate HEM formation by showing apparent elemental homogeneity. However, several studies have shown that small compositionally segregated domains within HEMs can go undetected by conventional STEM because its imaging mechanism averages the signal over the top 5–10 nm of the nanostructure, limiting surface sensitivity.

**Figure 3:**
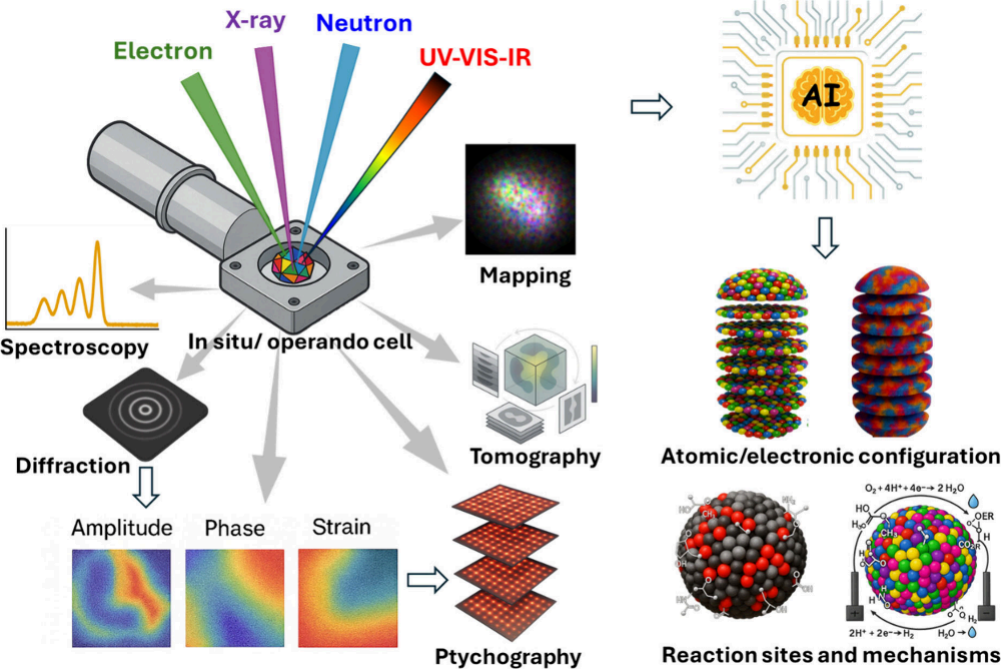
AI-aided correlative multimodal characterization. Schematic illustration of an AI-assisted multimodal characterization system integrating imaging and spectroscopy data to determine atomic organization, coordination environment, and electronic structure in HEMs, and to track active sites and mechanisms in an operando cell.

Likewise, probe-based infrared (IR) spectroscopy of reaction sites in diffuse reflectance IR Fourier transform spectroscopy (DRIFTS) mode and temperature-programmed desorption (TPD) can quantitatively determine site types, but inherently average over ensembles, failing to distinguish competing configurations. While useful for assessing HEM properties and functionality, they seldom resolve specific atomic configurations. Similarly, low-energy ion scattering (LEIS) probes the outermost atomic layer, and time-of-flight secondary ion mass spectrometry (ToF-SIMS) yields ultrashallow composition, yet both still provide ensemble-averaged top-layer information.

Dominant configurations can be inferred with techniques sensitive to surface elemental electronic structure and coordination, such as angle-resolved X-ray photoelectron spectroscopy (ARXPS), X-ray photoelectron spectroscopy (XPS), grazing-incidence X-ray absorption spectroscopy (GI-XAS), including X-ray absorption near-edge structure (XANES) and extended X-ray absorption fine structure (EXAFS). However, these still lack sufficient spatial resolution. Scanning tunneling microscopy (STM), including electrochemical STM (EC-STM), can directly visualize atomic distributions, though only in sufficiently flat facets.

Pushing spatial and chemical resolution beyond current limits is essential to obtain quantitative elemental maps that resolve short-range order, oxidation and coordination states, and adsorbates, thereby grounding robust structure/composition–property relationships. To this end, we need to integrate advanced electron microscopy workflows with spectroscopy techniques and artificial intelligence (AI)-assisted data analysis^49,50^ and denoising,^51^ spectral unmixing, structural recognition, 3D atomic modeling,^52^ and multimodal data fusion.^53^

STEM, using high-angle annular dark-field (HAADF), annular bright-field (ABF), or integrated differential phase contrast (iDPC), remains central to this precise characterization: HAADF provides Z-contrast for direct visualization of atomic columns and elemental discrimination, while ABF/iDPC offer complementary phase/low-Z sensitivity. Yet to push beyond current limits, further development is needed. In this direction, emerging four-dimensional (4D)-STEM techniques, particularly electron ptychography, are gaining traction. Ptychography is a computational imaging technique that scans a coherent beam (electrons, X-rays, light) over overlapping regions, records the resulting diffraction patterns, and uses phase-retrieval algorithms to reconstruct a high-resolution, quantitative image, often beyond lens limits. In particular, electron ptychography enables layer-by-layer reconstruction of nanostructures with record lateral resolution below 15 pm^54^ and significantly enhanced depth resolution compared to conventional STEM. Although current implementations do not yet provide true atomic-layer in-depth resolution, the technique shows strong potential for full 3D atomic reconstruction.

Moreover, electron ptychography offers the potential to resolve light elements such as oxygen and nitrogen, and even hydrogen,^55^ while enabling the localization of atomic vacancies and point defects,^56^ and quantifying near-surface strain linked to ordering. If future developments in electron ptychographic reconstruction algorithms, particularly those optimized through AI, succeed in achieving true atomic-scale resolution along the axial direction, this 3D-capable, element-specific surface-sensitive technique could directly map 3D element distributions and oxidation/valence states in complex materials such as HEMs. Such capability would provide exactly the kind of information needed to tackle fundamental questions in HEM electrocatalysis.

The rapid evolution of transformative 4D-STEM-based electron ptychography, coupled with the development of new AI-optimized algorithms, enabled by advances in computing power, including next-generation GPUs and cloud supercomputing directly linked to electron microscopes, is also pushing analysis toward real-time or near-real-time feedback. This will enable true in situ/operando experiments with 3D, atomic-resolution readouts while the system evolves, heralding a future in which atomic configurations and electronic structure can be tracked directly during operation to bridge theory and experiment.

Characterizing HEMs under working conditions, where potential/current, electrolyte, temperature, and reactants continually reshape the surface, is extremely challenging yet essential given their dynamic nature. This demands combined techniques and purpose-built operando liquid cells that provide stable referencing and thin diffusion layers, enabling correlative STEM and XAS characterization with frame-aligned electrochemical signals, complemented by spectro-electrochemical IR/Raman to follow adsorbates and reconstructions over time and potential. As support, rapid cryogenic quenching can “freeze” metastable states for subsequent ex-situ cross-validation.

In complex, compact electrochemical systems such as batteries, these challenges are amplified because the catalyst typically operates inside a sealed, multilayer, porous composite electrode (including binder/carbon/electrolyte and evolving solid products), often coupled to gas consumption/evolution and steep local gradients.^57^ As a result, operando access is constrained by cell geometry and signal attenuation, stable referencing is more difficult, and measurements can be strongly affected by parasitic reactions, electrolyte degradation, and dynamic product deposition that masks active sites and alters transport.

Beyond determining atomic organization in the as-synthesized HEM and tracking surface reorganization in operando conditions, identifying the truly active sites is essential to reveal mechanisms and design optimized compositions. Operando probes, XAS to follow changes in oxidation state/coordination, and FTIR to follow adsorbate fingerprints, can attribute activity to specific elements, while a broad set of reference samples can anchor these assignments. Yet even in the best case, such evidence typically reveals which element is active, not which of its ∼300,000 possible surface atomic configurations is responsible. Identifying the active configuration requires not only precise synthetic control that yields reference materials with deliberately tuned local ensembles as described above, but also ab initio density functional theory (DFT) combined with molecular dynamics (MD) and Monte Carlo (MC) simulations, which also face notable challenges when applied to complex, multielement, a priori randomly distributed materials.^15,58,59^

Ultimately, the immense compositional space is both the main appeal of HEM research and its greatest challenge. Surveying the vast range of possible compositions demands systematic, combinatorial, and automated synthesis combined with high-throughput screening to explore the space experimentally; high-throughput computation to predict phase stability, preferential coordination, electronic configurations, and performance; robust composition/processing–structure–property correlation capabilities; and the definition of proper physics-informed descriptors (Figure 4).^59–68,68–70^ In this regard, HEMs are an ideal testbed for data-driven materials discovery, where computation, experimentation, and AI converge to transform combinatorial diversity into actionable knowledge.

**Figure 4:**
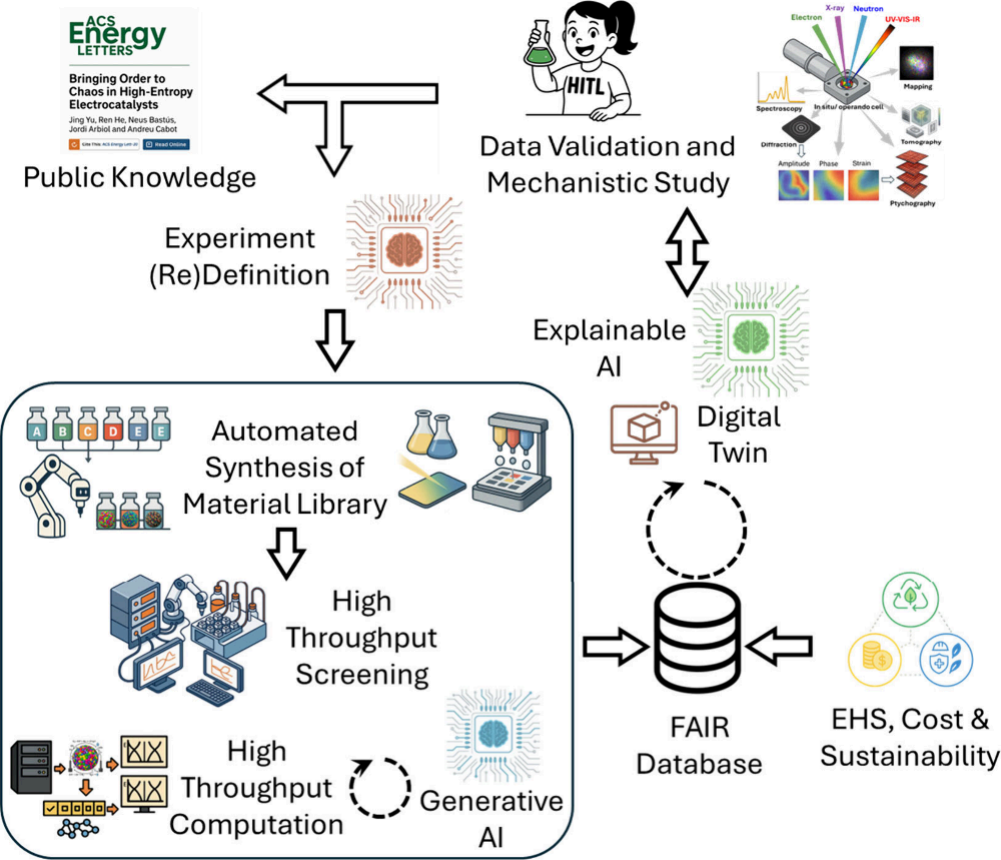
AI-driven HEM discovery platform. Closed-loop workflow integrating XAI with automated sample preparation, characterization, and simulation, feeding a findable, accessible, interoperable, and reusable (FAIR) database, with additional inputs from environmental, health, and safety (EHS), cost, and sustainability assessments.

Current online materials platforms cover only a tiny sliver of the HEM landscape relative to the immense combinatorial design space. Hence, there is a pressing need to build reliable, interoperable HEM libraries that couple experimental and computational data.

Experimentally, libraries should be built via composition-spread approaches (e.g., codeposition, droplet printing) and/or robotized batch or flow syntheses that systematically vary precursor types/ratios and process parameters (temperature, time, additives, concentrations). Automated post-treatments, such as annealing under controlled atmospheres and temperatures, should also be included as independent variables. Each parameter adds an axis to the multidimensional discovery space, reinforcing the need for automation and advanced data management.

Library screening should focus primarily on performance, using multiplexed, multimodal electrochemical cells with automated sample handling and electrolyte parameter control. Crucially, the right descriptors must be used, those that truly capture activity, selectivity, and stability under relevant conditions, quantified by cyclic voltammetry, impedance spectroscopy, and/or constant-current/constant-potential protocols, combined with online gas/liquid product analysis, including elemental dissolution monitoring. Inline IR, UV–vis, and Raman spectroscopy can be used to track intermediates, oxide/adsorbate layer formation, and surface reconstruction, yielding a multisignal fingerprint for each composition-process point.

In parallel, on the modeling side, DFT together with MD calculations should map HEM single-phase stability, formation energies, preferential configurations, and lattice strain under synthesis-relevant and operating conditions, along with d-band centers, work functions, adsorbate binding at realistic coverages, and charge-transfer under bias. To span the composition–structure space, thousands of HEM surface models with varied atomic arrangements should be assembled for each system. MD snapshots should feed back into DFT, enabling in-silico structural simulations directly comparable to experiment. Because exhaustive first-principles sampling is intractable, physics-informed, uncertainty-aware surrogate and generative machine learning (ML) models should interpolate across uncalculated configurations.

Data sets linking material parameters, processing conditions, experimental characterization results, and computational outputs should feed causality-aware explainable AI (XAI) algorithms. Interpretable learners then quantify how composition, structure, chemistry, electronic features, and processing govern activity, selectivity, and durability across a unified experimental–computational data set. To reach beyond what is physically synthesized, a digital experimental twin, trained on the aggregated multimodal data set, can simulate additional synthesis–characterization scenarios under realistic conditions, incorporating the learned causal relationships to define an appropriate exploration space. In this paradigm, AI is not a black-box fitting tool, but a scientific collaborator, uncovering causality and generating testable hypotheses for targeted validation.

In parallel, safe-and-sustainable-by-design (SSbD) considerations must be treated as first-class design variables and embedded directly within discovery workflows, rather than applied as post hoc evaluation criteria. SSbD, originally articulated as a voluntary framework to guide innovation in chemicals and materials, provides a structured logic for integrating safety and sustainability at early stages of materials development.^71^ In practice, SSbD is implemented through constraints (e.g., exclusion of toxic or critical elements and unsafe processing protocols) and secondary objectives (e.g., minimization of embodied energy, solvent burden, and predicted environmental, health, and safety risk), ensuring that each iteration jointly optimizes not only performance but also sustainability. By incorporating quantitative SSbD-relevant metrics, such as elemental criticality indices, toxicity and persistence, leaching and dissolution risks under electrochemical bias, synthesis process mass intensity, energy intensity, and end-of-life recyclability, AI-driven exploration is steered toward electrocatalyst compositions that are not only performant under laboratory conditions but also compatible with scale-up, long-term operation, and resource-constrained energy systems.

From the found correlations, and considering environmental, health, safety, cost, and sustainability parameters, the system should identify optimal configurations, rank candidates, and, crucially, (re)­define experimentally and computationally accessible descriptors to streamline both workflows.

Finally, verification is essential: predicted top candidates must be produced, benchmarked under standardized protocols, and interrogated operando to determine with precision the real working surfaces, reaction mechanisms, and reaction sites, as described above. Additionally, data from accelerated stress tests, long-term cycling, and post-mortem surface analyses can feed discrepancies back to models, continuously updating priors and refining acquisition policies.

As an additional layer, a fully automated, closed-loop autonomous discovery system can iteratively redefine what to make and how to make it, tightening synthesis protocols and constraining composition space over successive cycles. Data from automated screens and validation flow into retrained surrogate models informed by DFT and MD, guided by XAI to identify causal features. Uncertainty-aware, multiobjective design-of-experiments, e.g., Bayesian optimization or causal experimental design, proposes the next batch of compositions, synthesis/processing conditions, and operating variables. Crucially, the loop updates not only model parameters but also the descriptor set and the targets of the characterization and computational systems. In such an integrated closed-loop HEM discovery platform, active-learning controllers must tune acquisition on the fly using data quality metrics (signal-to-noise, spectral relevance), while predictive-maintenance and anomaly detection safeguards keep high-throughput operation reliable. Automated validation/conditioning routines and dynamic recalibration harden the pipeline against noise, drift, and equipment faults. The output is an interpretable, uncertainty-calibrated, multiobjective landscape (activity–stability–cost) that yields clear design rules and a prioritized, synthesizable short-list of candidate material synthesis and processing parameters that result in HEMs with optimized performance toward a particular electrocatalytic reaction.

Importantly, in the context of AI-assisted discovery of HEM electrocatalysts, model architectures and workflows must be designed to respect the underlying thermodynamic, electronic, and kinetic complexity of these materials rather than treating them as generic high-dimensional regression problems. In practice, this implies (i) deliberate selection of algorithms, ranging from uncertainty-aware Bayesian or Gaussian process models for data-scarce regimes to physics-informed neural networks and graph-based architectures for composition–structure–property learning; and (ii) the construction of descriptor sets that encode relevant quantities such as mixing enthalpy, configurational entropy, electronic structure proxies, adsorption energetics, and process variables, instead of naively concatenating all available experimental and computational parameters into a single feature vector. Such physically grounded featurization, together with dimensionality reduction, regularization, and rigorous cross-validation, helps mitigate overfitting and enables the extraction of chemically interpretable trends rather than purely correlative fits. Furthermore, model interpretation tools, e.g., sensitivity analysis, feature importance metrics, or symbolic regression layers, can be used to identify nontrivial structure–property relationships that can be fed back into mechanistic hypotheses about active sites, local environments, and degradation pathways in HEMs.

Achieving this level of rigor typically requires tight, iterative collaboration between materials scientists and ML practitioners: domain experts define meaningful target labels and constraints, critically evaluate whether learned correlations are physically plausible, and design validation experiments, while ML experts adapt model classes, loss functions, and uncertainty quantification schemes to the noise characteristics and biases inherent in high-throughput HEM data sets. Such bidirectional integration of domain knowledge and algorithm development is indispensable if AI tools are to move beyond black-box screening and deliver robust, generalizable design rules in the vast compositional landscape of high-entropy electrocatalysts.

Critically, to make the ecosystem broadly effective, the community should adopt shared data schemas, standardized formats, metadata conventions, and open benchmarks, ensuring reproducibility, comparability, and model transferability across laboratories. This coordination will turn HEM research from fragmented efforts into a cohesive, collaborative discovery network that accelerates innovation through collective intelligence.

Overall, realizing the promise of HEM electrocatalysis, including metal alloys, oxides, chalcogenides, phosphides, etc., demands rational design and precise engineering that fully exploit the compositional landscape to optimize performance. This requires beyond the state-of-the-art synthesis strategies with atomic distribution control and correlative multimodal characterization with advanced data processing capable of resolving not only surface composition and oxidation states ex situ but also their dynamic evolution in operando. A particularly challenging problem is pinpointing the true reaction-site configuration among thousands of possibilities and tuning composition, synthesis, and processing parameters to maximize the density of those sites. Equally critical is establishing a closed-loop discovery pipeline that seamlessly integrates automated synthesis, high-throughput experimentation, physics-based computation, and interpretable AI. A central challenge is reconciling the need for precise synthesis and rigorous characterization with the speed and breadth required for high-throughput materials discovery. When realized, this framework will narrow the gap between hypothesis and realization, accelerating the design cycle for next-generation catalysts. Beyond immediate applications in electrocatalysis, this integrative approach will reshape how materials are conceived, characterized, and optimized. The methodology is inherently transferable to other compositionally complex systems, paving the way for data-rich, AI-assisted, and ultimately autonomous materials research at scale. In this sense, HEMs serve not only as a versatile platform for catalysis but also as a proving ground for a new era of intelligent materials discovery.
